# PTC-209 Anti-Cancer Effects Involved the Inhibition of STAT3 Phosphorylation

**DOI:** 10.3389/fphar.2019.01199

**Published:** 2019-10-21

**Authors:** Shahrazad Sulaiman, Kholoud Arafat, Rabah Iratni, Samir Attoub

**Affiliations:** ^1^Department of Pharmacology and Therapeutics, College of Medicine and Health Sciences, United Arab Emirates University, Al-Ain, United Arab Emirates; ^2^Department of Biology, College of Science, United Arab Emirates University, Al-Ain, United Arab Emirates; ^3^Institut National de la Santé et de la Recherche Médicale (INSERM), Paris, France

**Keywords:** PTC-219, Frondoside-A, cisplatin, Bmi-1, STAT3

## Abstract

**Introduction:** Lung, breast, and colorectal cancers are the leading causes of cancer-related deaths despite many therapeutic options, including targeted therapy and immunotherapies.

**Methods:** Here, we investigated the impact of PTC-209, a small-molecule Bmi-1 inhibitor, on human cancer cell viability alone and in combination with anticancer drugs, namely, cisplatin, oxaliplatin, 5-fluorouracil, camptothecin, and Frondoside-A and its impact on cellular migration and colony growth *in vitro* and on tumor growth *in ovo*.

**Results:** We demonstrate that PTC-209 causes a concentration- and time-dependent decrease in the cellular viability of lung cancer cells (LNM35 and A549), breast cancer cells (MDA-MB-231 and T47D), and colon cancer cells (HT-29, HCT8/S11, and HCT-116). Similarly, treatment with PTC-209 significantly decreased the growth of LNM35, A549, MDA-MB-231, and HT-29 clones and colonies *in vitro* and LNM35 and A549 tumor growth in the *in ovo* tumor xenograft model. PTC-209 at the non-toxic concentrations significantly reduced the migration of lung (LNM35 and A549) and breast (MDA-MB-231) cancer cells. Moreover, we show that PTC-209, at a concentration of 1 μM, enhances the anti-cancer effects of Frondoside-A in lung, breast, and colon cancer cells, as well as the effect camptothecin in breast cancer cells and the effect of cisplatin in lung cancer cells *in vitro*. However, PTC-209 failed to enhance the anti-cancer effects of oxaliplatin and 5-fluorouracil in colon cancer cells. Treatment of lung, breast, and colon cancer cells with PTC-209 (1 and 2.5 μM) for 48 h showed no caspase-3 activation, but a decrease in the cell number below the seeding level suggests that PTC-209 reduces cellular viability probably through inhibition of cell proliferation and induction of cell death *via* a caspase-3–independent mechanism. Molecular mechanism analysis revealed that PTC-209 significantly inhibited the STAT3 phosphorylation by decreasing the expression level of gp130 as early as 30 min post-treatment.

**Conclusion:** Our findings identify PTC-209 as a promising anticancer agent for the treatment of solid tumors either alone and/or in combination with the standard cytotoxic drugs cisplatin and camptothecin and the natural product Frondoside-A.

## Introduction

In the last decade, the survival rate of cancer patients has improved with the application of targeted drugs, but despite these advances, cure of cancer remains a serious challenge (Torre et al., 2016). Nowadays, lung, breast, and colorectal cancers are the leading causes of cancer-related deaths, despite many therapeutic options, including targeted therapies and immunotherapies.

Bim-1, the polycomb group protein B lymphoma MoMLV Insertion Region 1, a component of the polycomb repressive complex 1 protein was shown to play an important role in tumorigenesis (Sauvageau and Sauvageau, 2010). In the recent years, the anti-cancer potential of PTC-209, a small specific inhibitor of Bmi-1, has been evaluated and showed efficacy, *in vitro* and *in vivo*, against various human cancers, such as colorectal (Kreso et al., 2014), ovarian (Dey et al., 2016), glioblastoma (Kong et al., 2018), head neck squamous cell carcinoma (Wang et al., 2017), multiple myeloma (MM) (Alzrigat et al., 2017), and chronic and acute myeloid leukemia (Nishida et al., 2015). PTC-209, which was originally identified as a low-molecular weight compound through high-throughput screening using gene expression modulation by small molecules technology (Kreso et al., 2014), was reported to exert its anticancer activity through specific targeting of Bmi-1 expression. Indeed, PTC-209 was able to downregulate the level of Bmi-1 transcript in the human colorectal HCT116 and fibrosarcoma HT1080 cancer cells (Kreso et al., 2014). Further studies showed that PTC-209 downregulated the level of Bmi-1 protein without affecting the levels of Bmi-1 transcript in MM cells (Wang et al., 2017) and biliary tract cancer (BTC) cells (Mayr et al., 2016), suggesting that it might also regulate Bmi-1 expression at the posttranscriptional level.

Here, we investigated the preclinical anticancer activity of PTC-209 against three major solid tumors, namely, lung, breast, and colon cancer either alone, or in combination with clinically approved cytotoxic drugs and the new natural compound, frondoside A.

## Materials and Methods

### Cell Culture and Reagents

Human lung cancer cells, LNM35 and A549, were maintained in RPMI 1640 (Hyclone, Cramlington, UK), human breast cancer cells, MDA-MB-231 and T47D; human colon cancer cells HT-29, HCT-116, and HCT8/S11; and mouse gastric stem cells (MGSC) were maintained in DMEM (Hyclone, Cramlington, UK). All media were supplemented with antibiotics (penicillin, 50 U/ml; streptomycin, 50 µg/ml) (Hyclone, Cramlington, UK) and with 10% foetal bovine serum (Hyclone, Cramlington, UK). In all experiments, cell viability was higher than 99% using trypan blue dye exclusion. The culture medium was changed every 3 days, and cells were passaged once a week when the culture reached 95% confluence. PTC-209 was purchased from Xcess Biosciences Inc (Xcess Biosciences Inc, San Diego, CA). Frondoside A, camptothecin, cisplatin, oxaliplatin, and 5-fluorouracil were purchased from Sigma-Aldrich (Saint Louis, MO). Antibodies to β-tubulin, gp130, STAT3, phospho-STAT3 were obtained from Cell Signaling Technology (Cell Signaling, Beverly, MA). Bmi-1 antibody was obtained from Abcam (Cambridge, UK). Antibody to β-actin was obtained from Santa Cruz Biotechnology, Inc (Santa Cruz, CA).

### Cellular Viability

Cells were seeded at a density of 5,000 cells/well into 96-well plates. After 24 h, cells were treated for another 24, 48, and 72 h with increasing concentrations of PTC-209 (0.01–10 µM) in triplicate. Control cultures were treated with 0.1% DMSO (the drug vehicle). The effect of PTC-209 on cell viability was determined using the CellTiter-Glo Luminescent Cell Viability Assay (Promega Corporation, Madison, WI), based on quantification of ATP, which signals the presence of metabolically active cells. The luminescent signal was measured using the GLOMAX Luminometer system. Data were presented as proportional viability (%) by comparing the PTC-209-treated cells with the DMSO-treated cells, the viability of which is assumed to be 100%.

In the second set of experiments, cells were treated for 48 h with a combination of PTC-209 and cisplatin, PTC-209 and camptothecin, PTC-209 and oxaliplatin, PTC-209 and 5-fluorouracil, or PTC-209 and Frondoside-A. The effects of these combinations on cell viability were presented as proportional cell viability (%) by comparing the drug-treated cells with the DMSO-treated cells, the viability of which is assumed to be 100%.

### BrdU Proliferation Assay

Cell proliferation bromodeoxyuridine (BrdU) assay obtained from Roche Biochemicals, Indianapolis, IN was used to determine the effect of PTC-209 on cell proliferation/number. Cells were seeded at a density of 5,000 cells/well into 96-well plates and after 24-h incubation (day 0), cells were treated for another 48 h with or without PTC-209 (1 and 2.5 µM) followed by a 2.5-h incubation with BrdU solution. BrdU was also measured at day 0 to determine the number of cells at time 0. The incubation and the reading procedure were performed as per the manufacturer’s instructions.

### Caspase 3/7 Activity

Cells were seeded at a density of 5,000 cells/well into 96-well plate and treated with PTC-209 (1 and 2.5 µM) for 48 h in triplicate. Control cells were exposed to DMSO 0.1%. Caspase-3/7 activity was measured using a luminescent Caspase-Glo 3/7 assay kit following the manufacturer’s instructions (Promega Corporation, Madison, WI). Caspase reagent was added, and the plate was mixed and incubated for 2.5 h at room temperature. Luminescence was measured using a GLOMAX Luminometer system.

### Clonogenic Assay

Cells were seeded into six-well plates at a density of 50 cells per well for A549 cells and 100 cells per well for LNM35, MDA-MB-231, and HT-29 cells. After 24 h, cells were treated for another 48 h with increasing concentration PTC-209 (0.1 to 2.5 µM) and cultured for an additional 14 days. Then, colonies were washed three times with PBS, fixed, and stained for 2 h with 0.5% crystal violet dissolved in (v/v) distilled water/methanol. Clones were washed three times with PBS, photographed, and counted. The percentages of colonies with more than 50 cells were determined and compared with the DMSO-treated colonies assumed to be 100%. The experiment was repeated three times.

### Colony Growth in Matrigel Matrix

A layer of 150 µl of matrigel was poured into the wells of a 24-well cell culture dish and allowed to set at 37°C for 30 min. A second layer (300 µl) composed of 150 µl of matrigel dissolved in 150 µl of growth media containing 1.5 × 10^3^ cells was placed on top of the first layer and allowed to set in the humidified incubator at 37°C for 30 min. Growth medium (0.5 ml) was added on top of the second layer, and the cells were incubated in a humidified incubator at 37°C for 14 days to form colonies and then treated for another 7 days with PTC-209 (1 and 2.5 µM). Control cells were exposed to 0.1% DMSO. The medium was changed twice a week. At the end of the experiment, colonies were stained for 1 h with 2% Giemsa stain and incubated with PBS overnight to remove excess Giemsa stain. Colonies were photographed and scored under an inverted microscope. Colonies that are equal to or larger than 200 µm were considered as large colonies and were expressed as a percentage of the total counted colonies in PTC-209–treated wells and compared with the DMSO-treated controls.

### *In Ovo* Tumor Growth Assay

Fertilized White Leghorn eggs were incubated at 37.5°C and 50% humidity for 9 days. At the embryonic day 9 (E9), the chorioallantoic membrane (CAM) was dropped by drilling a small hole through the eggshell into the air sac, and a 1-cm^2^ window was cut in the eggshell above the CAM. Cancer cells were trypsinized, washed with complete medium, and suspended in PBS. A 50-µl inoculum of 1 × 10^6^ cells was added onto the CAM of each egg, for a total of 10 to 15 eggs per treatment condition (to get sufficient surviving embryos at the end of the experiments). Two days later, tumors that began to be detectable were treated every second day at E11, E13, E15, and E17 by dropping 100 μl of the vehicle (PBS with 0.1% of DMSO) or PTC-209 (5 µM). At E18, the upper portion of the CAM was removed, washed in PBS, and then the tumors were carefully cut away from normal CAM tissues and weighted to determine the impact of PTC-209 on tumor growth. The *in ovo* LNM35 xenografts assay was done according to the protocol approved by the animal ethics committee at the United Arabs Emirates University. The *in ovo* A549 xenografts assay was done by INOVOTION company in France. The eggs were randomly assigned to the treatments, but the experimenter was not blinded to the identities of the groups. All data collected were used in statistical analysis. According to the European Directive 2010/63/EU on protection of animals used for scientific purposes and French Regulations (Code Rural R214-89 to R214-137, last modification in 2013) which cover the use of chicken embryos at day 18 post-fertilization or later, there are no ethic constraints because our studies were stopped on day 18 of the embryo development (E18). Moreover, the Animal Experimentation Ethical Comity of Grenoble area has validated that we do not need Institutional Animal Care and Use Committee (IACUC) approvals for our assays.

### Impact of PTC-209 on Cellular Migration Using Wound Healing Assay

LNM35, A549, and MDA-MB-231 cells were grown in six-well tissue culture dishes until confluence. A scrape was made through the confluent monolayer with a plastic pipette tip of 1-mm diameter. Afterward, the dishes were washed twice and incubated at 37°C in fresh medium containing 10% foetal bovine serum and two non-toxic concentration of PTC-209 (0.01–0.1 µM). At the bottom side of each dish, two arbitrary places were marked where the width of the wound was measured with an inverted microscope (objective, ×4) (Olympus 1X71, Japan). Migration was expressed as mean ± SEM of the difference between the measurements at time 0 and the 2-, 6-, and 24-h periods considered. Each experiment was repeated at least three times.

### Western Blotting Assay

A549 and MDA-MB-231 cells were seeded in 100-mm dishes at 2 × 10^6^ cells/dish for 24 h and then treated with two concentrations of PTC-209 (1 and 2.5 µM) for another 0.5, 2, 6, 24, and 48 h. Control cultures were treated with 0.1% DMSO (the drug vehicle). Total cellular proteins were isolated using RIPA buffer (25 mM Tris-HCl, pH 7.6, 1% nonidet P-40, 1% sodium deoxycholate, 0.1% SDS, 0.5% protease inhibitors cocktail, 1% PMSF, 1% phosphatase inhibitor cocktail) from DMSO- and drugs-treated cells. The whole cell lysates were recovered by centrifugation at 14,000 rpm for 20 min at 4°C to remove insoluble material, and protein concentration of lysates were determined by BCA protein assay kit (Thermo Fisher Scientific, Waltham, MA). Proteins (30 µg) were separated by SDS-PAGE gel for the expression of gp130, STAT3 and the phosphorylation level of STAT3. Proteins from LNM35, A549, MDA-MB-231, T47D, HT-29, HCT-116, and HCT8/S11 were also analyzed for the expression of Bmi-1, STAT3, and Phospho-STAT3. After electrophoresis, the proteins were transferred onto a nitrocellulose membrane; blocked for 1 h at room temperature with 5% non-fat milk in TBST (TBS and 0.05% Tween 20); and then probed with specific primary antibodies, β-tubulin, and β-actin overnight at 4°C. The blots were washed and exposed to secondary antibodies. Immunoreactive bands were detected using ECL chemiluminescent substrate (Thermo Fisher Scientific), and chemiluminescence was visualized using the LiCOR C-DiGit blot scanner (LI-COR Biotechnology, US). Membrane stripping was performed by incubating the membrane in Restore Western blot stripping buffer (Thermo Fisher Scientific) according to the manufacturer’s instructions. Densitometry analysis was performed using an HP Deskjet F4180 Scanner with ImageJ software. The intensities of the bands were normalized to the intensities of the corresponding β-actin bands.

### Statistics

Each experiment was repeated at least three times except the *in ovo* assay. Results were expressed as means ± SEM of the indicated data. The difference between experimental and control values was assessed by ANOVA followed by Dunnett’s multiple comparisons test. For the combination experiments, data were assessed by ANOVA followed by Tukey’s multiple comparisons test. ****P < 0.0001, ***P < 0.001, **P < 0.01, and *P < 0.05 indicate significant differences.

## Results

### PTC-209 Decrease Cellular Viability and Colonies Growth *In Vitro* and Tumor Growth *In Vivo*

**Figure 1:**
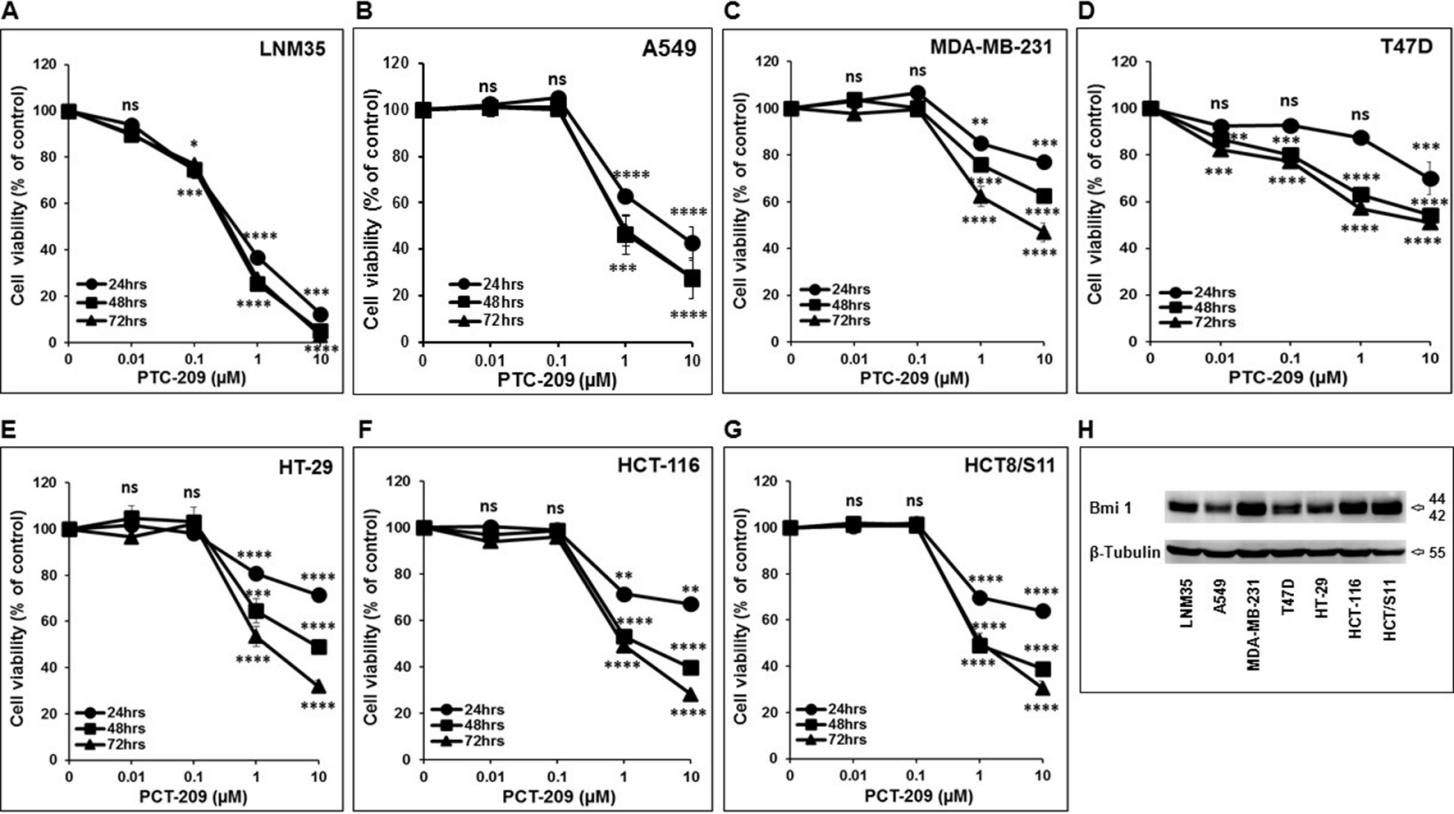
Inhibition of cellular viability by PTC-209. Exponentially growing LNM35 **(A)**, A549 **(B)**, MDA-MB-231 **(C)**, T47D **(D)**, HT-29 **(E)**, HCT-116 **(F)**, HCT8/S11 **(G)** cells were treated with vehicle (0.1% DMSO) and the indicated concentrations of PTC-209 for 24, 48, and 72 h. Viable cells were assayed as described in Materials and Methods. **(H)** Western blot analysis of Bmi-1 protein level expression in LNM35, A549, MDA-MB-231, T47D, HT-29, HCT-116, and HCT8/S11 cells. β-tubulin was used as a loading control. All experiments were repeated at least three times. Shapes represent means; bars represent S.E.M. *Significantly different at *P* < 0.05, **Significantly different at *P* < 0.01, ***Significantly different at *P* < 0.001, ****Significantly different at *P* < 0.0001, ns (not significant).

We have investigated the impact of increasing concentration of PTC-209 (0.01–10 µM) on various cancer cell lines, namely, lung (LNM35, A549), breast (MDA-MB-231 and T47D), and colon (HT-29, HCT-116, and HCT8/S11). As shown in **Figure 1**, PTC-209 induced a concentration- and time-dependent decrease in the cellular viability of all cell lines tested (**Figures 1A–G**). These cells express moderate differences in the expression levels of Bmi-1 (**Figure 1H**).

**Figure 2:**
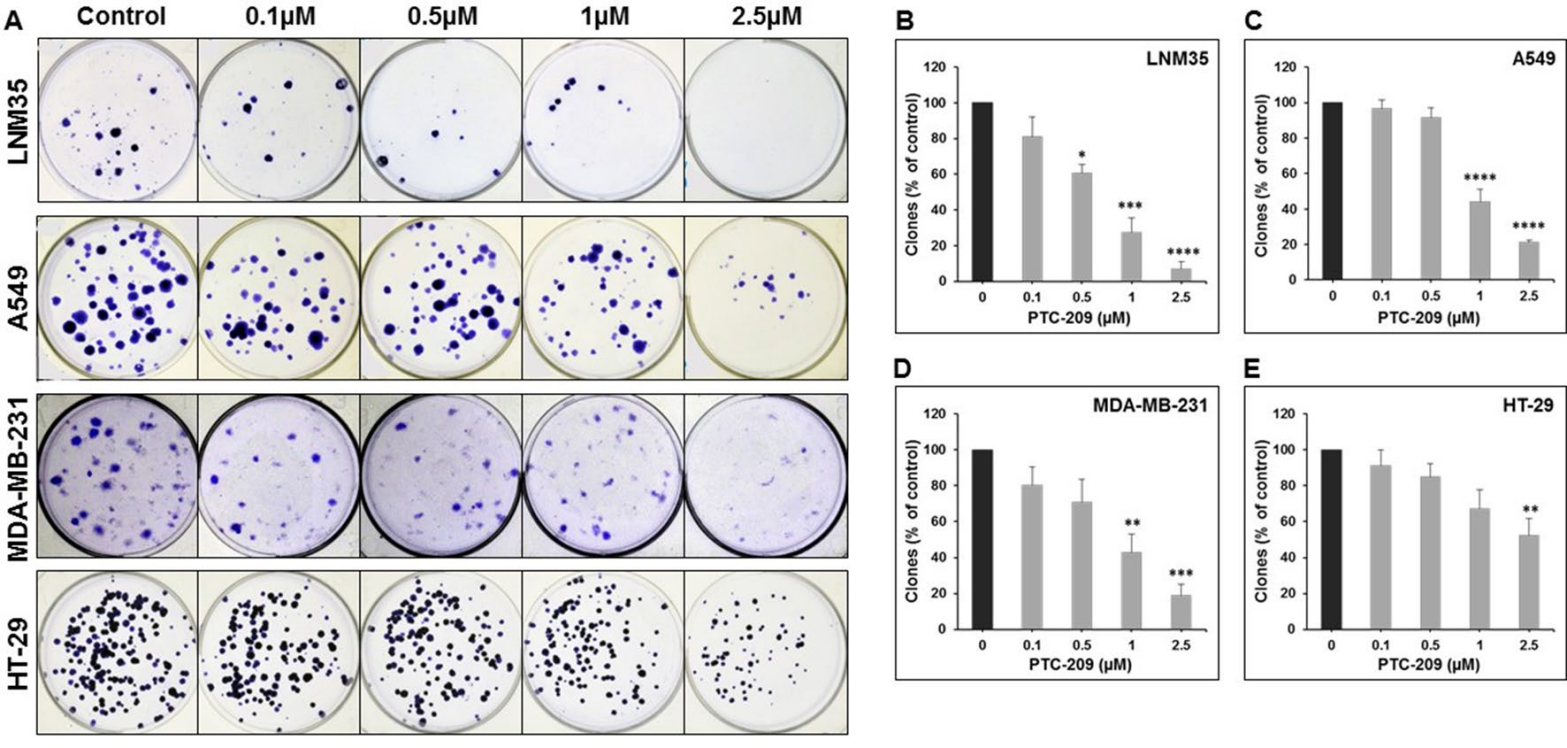
PTC-209 inhibits the clonogenic growth of lung, breast, and colon cancer cell lines. **(A)** Lung (LNM35 and A549), breast (MDA-MB-231), and colon (HT-29) cancer cells were grown in the presence or absence of increasing concentration of PTC-209 for 48 h and then incubated for 14 days to form colonies. **(B**–**E)** The inhibition of the growth of cancer cell-derived colonies was assessed by measuring the number of the colonies obtained in control and PTC-209-treated wells as described in Materials and methods. Data represent the mean of three independent experiments. *Significantly different at *P* < 0.05, **Significantly different at *P* < 0.01, ***Significantly different at P < 0.001, ****Significantly different at P < 0.0001.

To further confirm the anticancer potential of PTC-209, we examined its ability to modulate the growth capacity of colonies formed using the clonogenic formation assay. Toward this, lung (LNM35 and A549), breast (MDA-MB-231), and colon (HT-29) cancer cells were grown in the presence or absence of increasing concentration of PTC-209 for 2 days and then cultured for an additional 14 days to form colonies. As shown in **Figure 2**, treatment with PTC-209 caused a concentration-dependent decrease in the number of colonies. In agreement with cell viability data, lung cancer cells (LNM35 and A549) showed a higher sensitivity to PTC-209 treatment (**Figures 2B, C**) compared with breast (MDA-MB-231) and colon (HT-29) cancer cells (**Figures 2D, E**).

**Figure 3:**
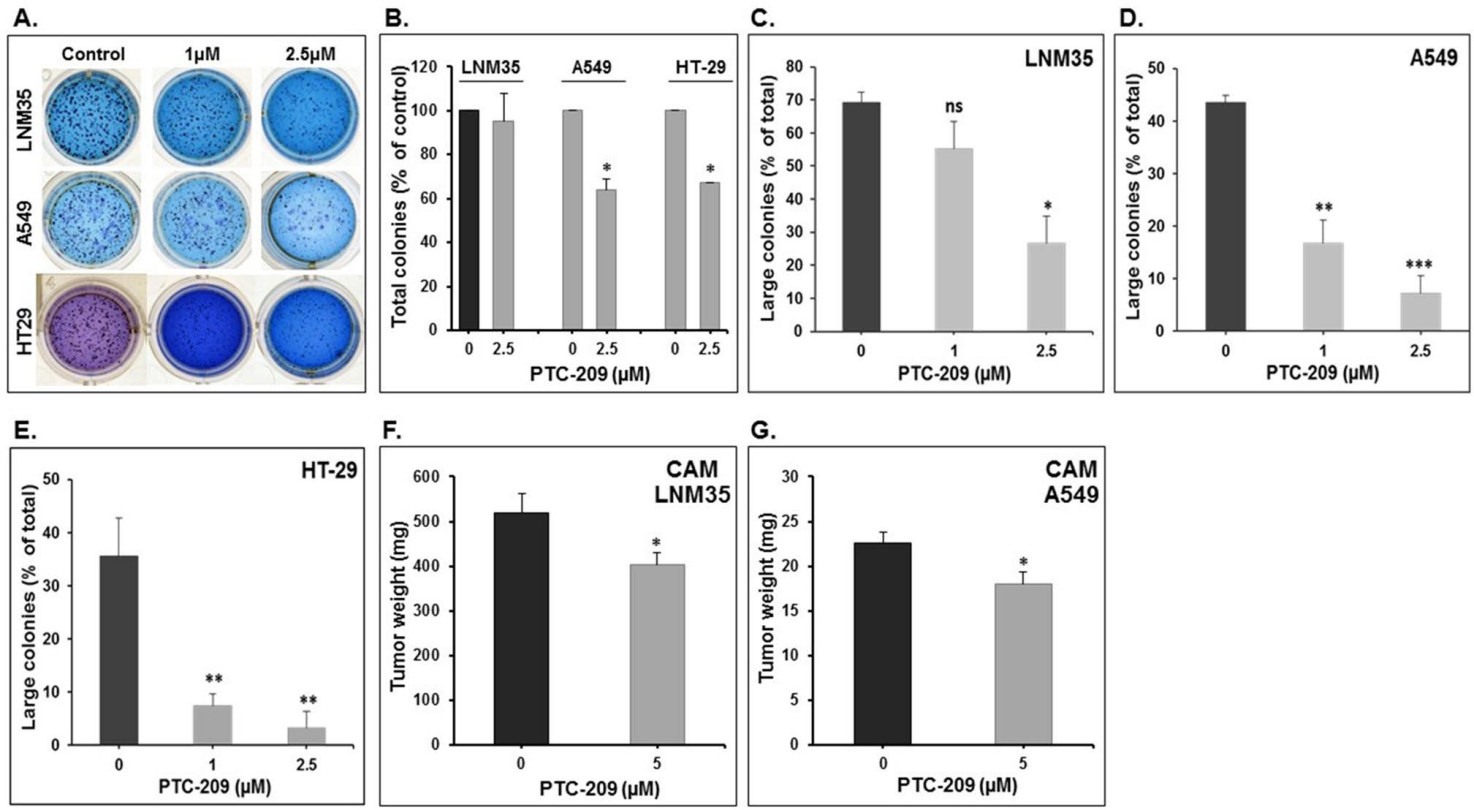
Effect of PTC-209 on the tri-dimension colonies growth and on tumor growth in the *in ovo* xenograft model. **(A)** Representative pictures showing the impact of PTC-209 (1 and 2.5 µM) on LNM35, A549, and HT-29 total colonies number in Matrigel matrix after 7 days of treatment. **(B)** Impact of PTC-209 (2.5 µM) on LNM35, A549, and HT-29 total colonies number. Inhibition of LNM35 **(C)**, A549 **(D)**, HT-29 **(E)** colonies growth. Data presented here are the mean percentage of large colonies (≥200 µm). All experiments were repeated at least three times. **(F)** LNM35 and **(G)** A549 cells (1 × 10^6^) were grafted on the CAM of 9-day (E9) chick embryo. Tumors were treated every 48 h with PTC-209 as described in Materials and Methods. At E18, tumors were collected and weighted. Columns are means; bars are S.E.M. *Significantly different at P < 0.05, **Significantly different at P < 0.01, ***Significantly different at P < 0.001. ns (not significant).

The anticancer potential of PTC-209 was also assessed by examining its effect on the growth of already-formed LNM35, A549, and HT-29 colonies in matrigel matrix assay. Cells were first allowed to form visible colonies for 2 weeks and then treated with or without 1- and 2.5-µM PTC-209 and allowed to grow for 1 more week. Although the total number of LNM35 colonies remained unaffected (5% decrease) by 2.5-µM PTC-209 treatment (**Figures 3A, B**), PTC-209, at a concentration of 2.5 µM, significantly reduced the total number of A549 and HT29 colonies by 36% and 33%, respectively (**Figures 3A, B**). Furthermore, the sizes of the colonies were dramatically reduced in PTC-treated compared with control plate, and this is regardless of the cancer cells tested (**Figures 3C–E**). These results clearly demonstrate that treatment with PTC-209 resulted in a significant decrease in the growth of the pre-established colonies. Taken together, these data strongly suggest that PTC-209 possess a broad anticancer spectrum activity *in vitro*.

To confirm the pharmacological relevance of our *in vitro* data, the anticancer activity of PTC-209 was investigated *in vivo* using the *in ovo* chick embryo tumor growth assay. Here, we focused our investigation on the A549 and LNM35 lung cancer cells. Cells grafted on the CAM formed tumors that were treated every 48 h with vehicle (DMSO), or PTC-209 (5 µM). At E18, tumors were recovered from the upper CAM and weighed. In line with our *in vitro* findings, we found that PTC-209 at a concentration of 5 µM significantly reduced the growth of LNM35 and A549 tumor xenografts by 22% and 20.2%, respectively (**Figures 3F, G**). Toxicity was also evaluated by comparing the number of dead embryos in control and PTC-209–treated groups. At the end of the experiment (E18), PTC-209 showed no cytotoxicity as there was no difference in the number of surviving embryo in control and PTC-209 treatment (data not shown).

### PTC-209 Exert Its Anticancer Activity Through Inhibition of Cellular Proliferation and Induction of Cell Death

**Figure 4:**
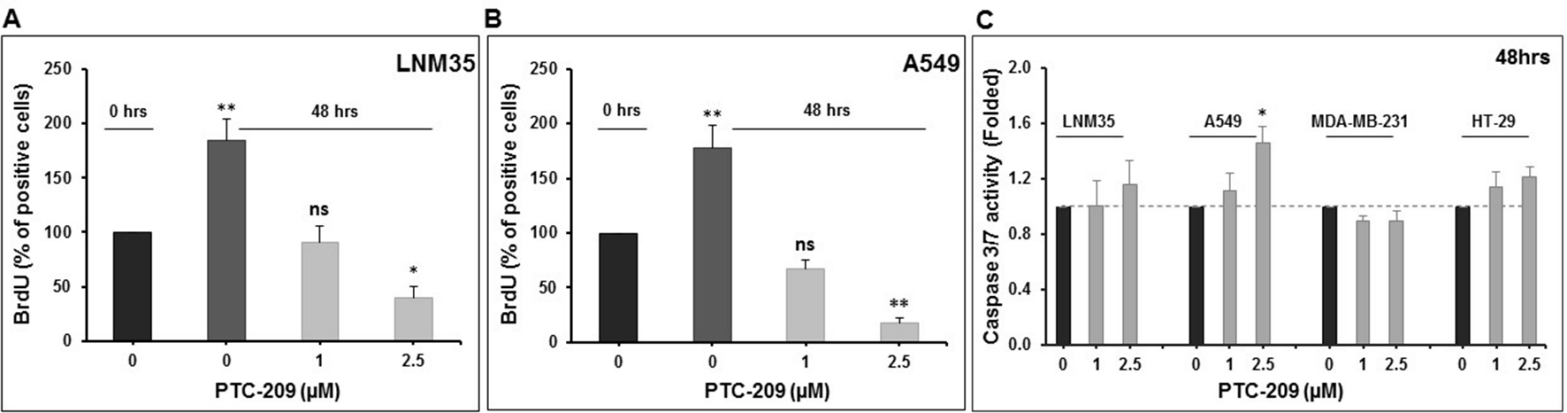
Effects of PTC-209 on cellular proliferation and caspase activation. **(A** and **B)** PTC-209 (1–2.5 µM) decreases cellular proliferation of LNM35 and A549, as measured by BrdU incorporation assay. **(C)** Caspase-3/7 activity was analyzed in LNM35, A549, MDA-MB-231, and HT-29 cells treated for 48 h with PTC-209 (1–2.5 µM), normalized to the number of viable cells per well and expressed as fold induction compared with the control group. Columns are means; bars are S.E.M. *Significantly different at *P* < 0.05. **Significantly different at *P* < 0.01, ns (not significant).

We have shown above that PTC-209 significantly inhibited cell viability and anchorage-dependent and anchorage-independent colony growth. Next, we sought to examine the mechanism(s) of inhibition (cellular proliferation, apoptotic cell death, or both) through which PTC-209 exerts its anticancer activity. Toward this, we first measured the proliferation level of control- and PTC-treated cells by measuring BrdU incorporation. Interestingly, we saw that the numbers of both LNM35 and A549 cells after 48 h treatment with PTC-209 were lower than the numbers of cells at 0 h hence, suggesting the occurrence of cell death upon PTC-209 treatment. Our results clearly show that PTC-209 (2.5 μM) not only significantly reduced the cellular proliferation of lung cancer cells but also induced cellular death (**Figures 4A, B**).

Because cell death is induced in PTC-treated cells, we sought to determine whether it involves the activation of the apoptotic pathway. For this, we determined the level of active caspase 3/7, a marker of apoptosis, in PTC-209-treated cells. Although a concentration of 1 and 2.5 μM, which dramatically reduced cell viability, had no effect on caspase 3/7 activation in all cancer cell lines tested, a mild but significant 1.46-fold increase in caspase 3/7 was seen in A549 cells treated with 2.5 μM of PTC-209 (**Figure 4C**). Taken together, our results strongly suggest that PTC-209 exerts its anticancer activity through the inhibition of cellular proliferation and the induction of cellular death presumably independent of caspase 3/7 mechanism.

### PTC-209 Impairs Cancer Cell Migration *In Vitro*

**Figure 5:**
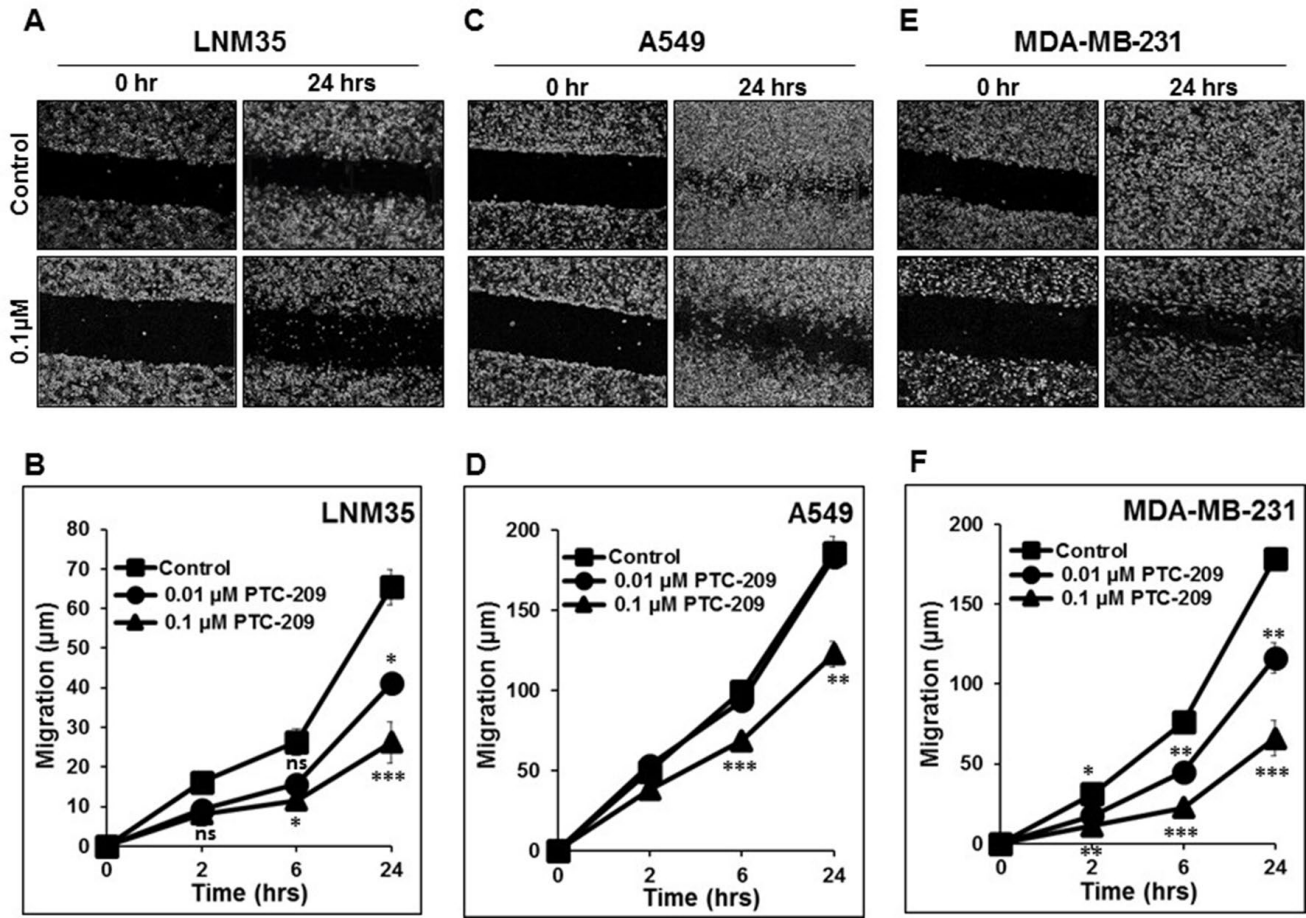
PTC-209 impairs cancer cell migration. Wounds were introduced in LNM35 **(A** and **B)**, A549 **(C** and **D)**, and MDA-MB-231 **(E** and **F)** confluent mono-layers cultured in the presence or absence (control) of PTC-209 (0.01 and 0.1 µM). The mean distance that cells travelled from the edge of the scraped area for 2, 6, and 24 h was measured in a blinded fashion, using an inverted microscope (4× magnifications). All experiments were repeated at least three times. Shapes represent means; bars represent S.E.M. *Significantly different at P < 0.05, **Significantly different at *P* < 0.01, ***Significantly different at *P* < 0.001, ns (not significant).

Cancer cell migration and invasion leading to metastasis are responsible of 90% of cancer-related death. In this context, we demonstrate that PTC-209 at the non-toxic concentration (0.01 and 0.1 µM) induced a time- and concentration-dependent inhibition of cellular migration of LNM35 (**Figures 5A, B**), A549 (**Figures 5C, D**), and MDA-MB-231 (**Figures 5E, F**).

### PTC-209 Decrease STAT3 Phosphorylation

**Figure 6:**
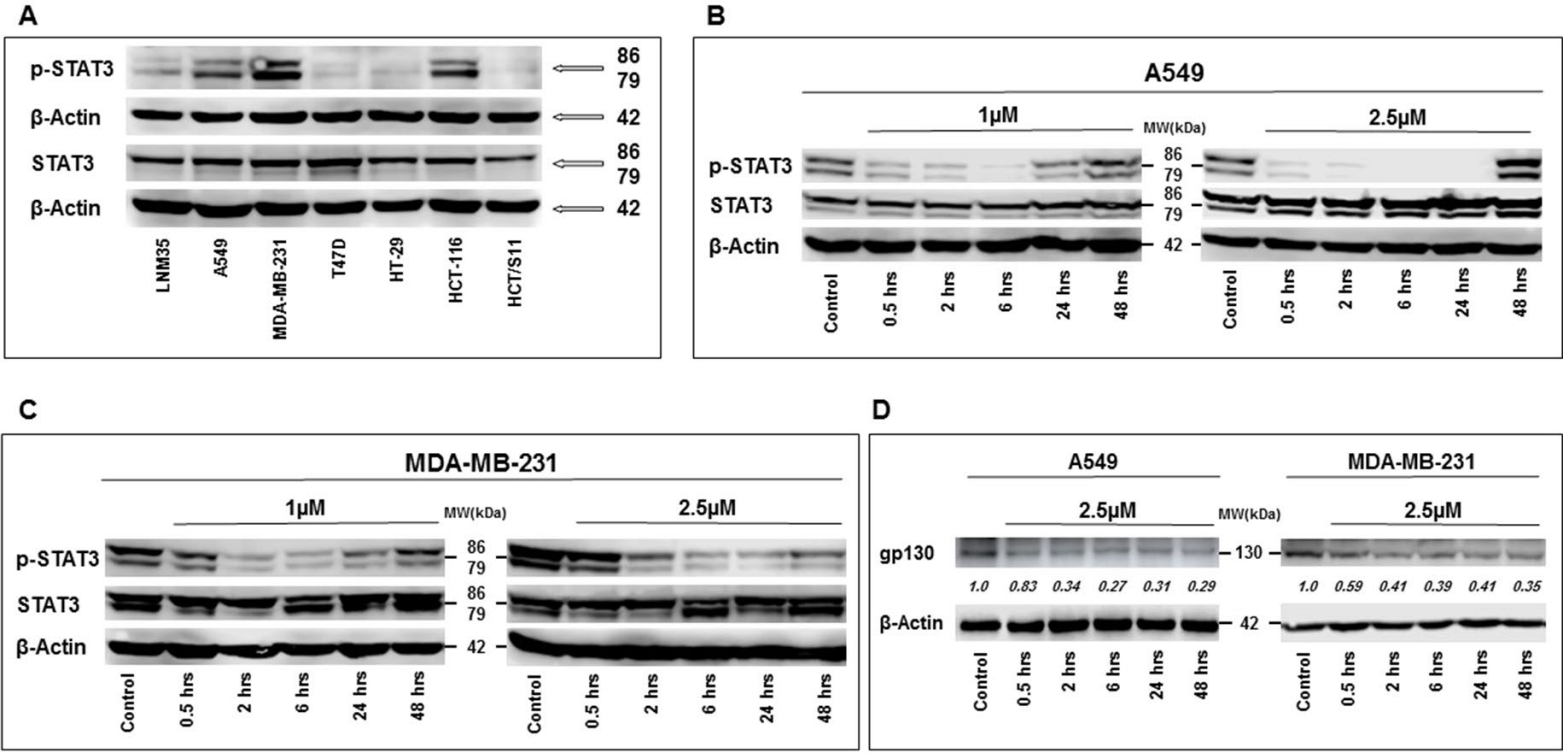
Western blot analysis of total and phosphorylated STAT3 expression levels in LNM35, A549, MDA-MB-231, T47D, HT-29, HCT-116, and HCT8/S11 cells **(A)**. Inhibition of STAT3 phosphorylation by PTC-209 in A549 lung cancer cells **(B)**, and MDA-MB-231 breast cancer cells **(C)**. Cells were treated with 1- and 2.5-μM PTC-209 and proteins were extracted at the indicated time-points (0.5, 2, 6, 24, and 48 h). **(D)** Decrease in gp130 expression in A549 and MDA-MB-231 cells treated with 2.5 μM of PTC-209. β-actin was used as a loading control. Numbers below each blot indicate changes in band intensity compared to control, as determined by densitometric analysis. Data are representative of three independent experiments.

The tyrosine phosphorylation of STAT3 is required for their dimerization, nuclear translocation, and DNA binding. It is well established that chronic activation/phosphorylation of STAT3 is associated with cellular transformation and tumor progression, including cellular proliferation and cellular migration and invasion. In this context, we examined whether the anti-cancer effect of PCT-209 involves the modulation of the STAT3 pathway. Toward this, we first determined the levels of activated (phosphorylated) STAT3 in LNM35, A549, MDA-MB-231, T47D, HT-29, HCT-116, and HCT8/S11 cells. STAT3 was clearly constitutively phosphorylated in A549, MDA-MB-231, and HCT-116 cells (**Figure 6A**). We decided to investigate the level of phosphorylated STAT3 over periods (0.5, 2, 6, 24, and 48 h) in the lung (A549) and breast (MDA-MB-231) cancer cells lines treated with 1 and 2.5 μM of PTC-209. As shown in **Figure 6**, PTC-209 significantly decreased the level of phosphorylated STAT3 in A549 (**Figure 6B**) and MDA-MB-231 (**Figure 6C**). This inhibition of STAT3 phosphorylation was observed as early as 30 min post-treatment at both concentrations used in either cell lines. This inhibition was maintained for almost 48 h. PTC-209 treatment had no effect on the level of total STAT3. Constitutive activation of STAT3 in A549 and MDA-MB-231 cells has been reported to be linked to an autocrine loop involving IL-6, IL-6R, gp130, and STAT3. In parallel to the inhibition of STAT3 phosphorylation, we observed that treatment of A549 and MDA-MB-231 cells with 2.5 μM of PTC-209 for 0.5, 2, 6, 24, and 48 h resulted in a rapid decrease of gp130 expression (**Figure 6D**). Hence, our findings strongly suggest that PTC-209 mediates its anti-cancer effect on several cancer cell lines, at least in part, through downregulation of STAT3 pathway and do not exclude the possible involvement of other signaling pathways in the inhibition of STAT3 phosphorylation by PTC-209.

### PTC-209 Enhances the Anticancer Activity of Cisplatin and Frondoside-A

In clinical practice, combination therapy is the main approach in the management of cancer. To further study the therapeutic potential of PTC-209, we investigated whether its anti-growth effects could enhance the anticancer activity of major chemotherapeutic drugs, namely, cisplatin on lung cancer cells; LNM35 and A549; camptothecin on breast cancer cells, MDA-MB-231; oxaliplatin and 5-fluorouracil on colon cancer cell, HT-29. In parallel, we investigated the impact of the combination PTC-209 and a natural triterpenoid glycoside, Frondoside A, on six different cell lines, namely, LNM35, A549, MDA-MB-231, HT-29, HCT-116, and MGSC.

**Figure 7:**
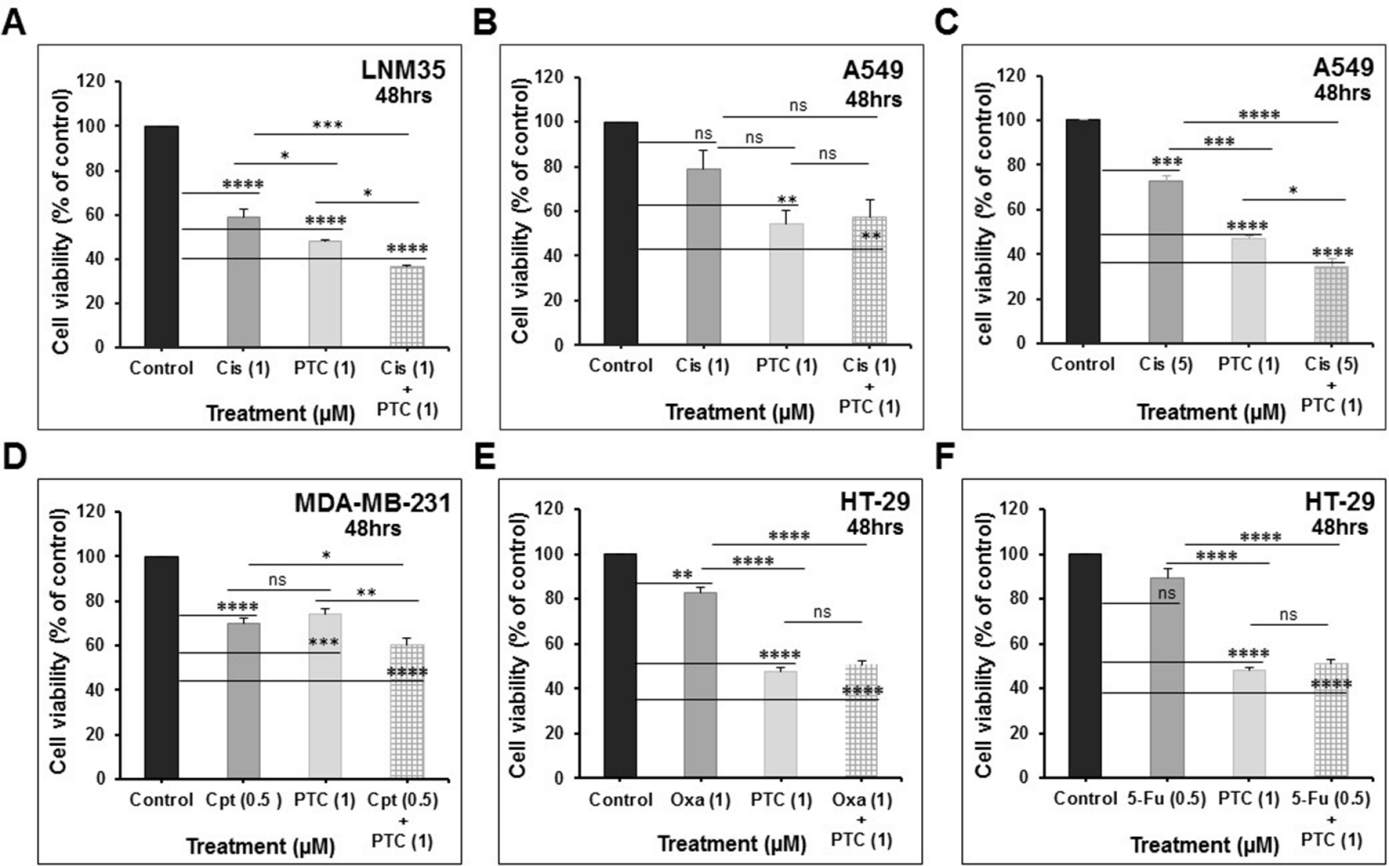
Impact of PTC-209 (1 μM) on the inhibition of cell viability by: **(A)** cisplatin (1 μM) in LNM35 cells, **(B** and **C)** cisplatin (1–5 μM) in A549 cells, **(D)** camptothecin (0.5 μM) in MDA-MB-231 cells, **(E)** oxaliplatin (1 μM) in HT-29 cells, and **(F)** 5-fluorouracil (0.5 μM) in HT29 cells. All experiments were repeated at least three times. Columns are means; bars are S.E.M. *Significantly different at *P* < 0.05, **Significantly different at *P* < 0.01, ***Significantly different at P < 0.001, ****Significantly different at *P* < 0.0001. ns (not significant). PTC-209 (PTC), cisplatin (Cis), camptothecin (Cpt), oxaliplatin (Oxa), and 5-fluorouracil (5-Fu).

**Figure 8:**
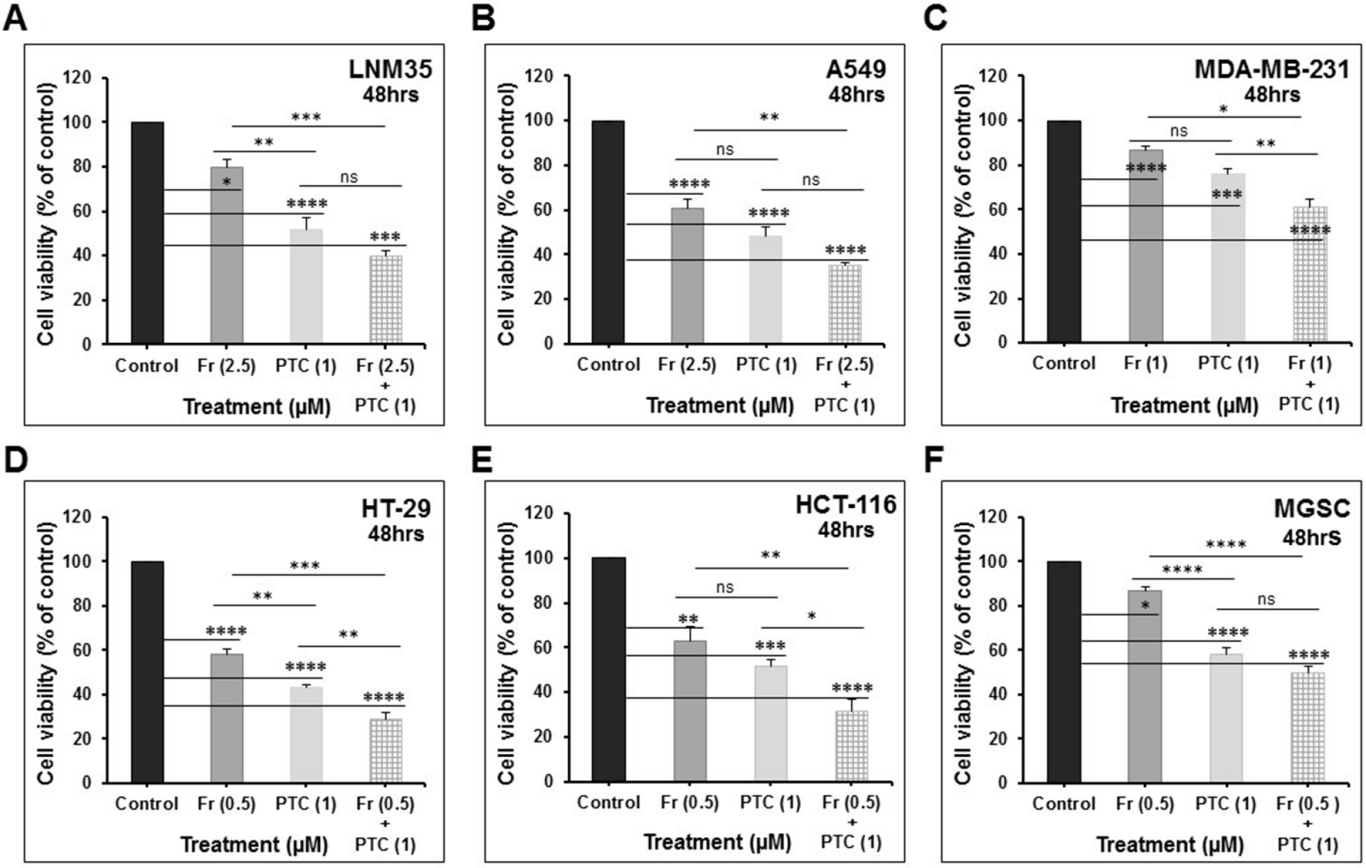
PTC-209 (1 μM) enhances the inhibition of cell viability by Frondoside-A (0.5–2.5 μM) in: **(A)** LNM35 cells, **(B)** A549 cells, **(C)** MDA-MB-231 cells, **(D)** HT-29 cells, **(E)** HCT-116 cells, and **(F)** MGSC cells. All experiments were repeated at least three times. Columns are means; bars are S.E.M. *Significantly different at *P* < 0.05, **Significantly different at *P* < 0.01, ***Significantly different at *P* < 0.001, ****Significantly different at *P* < 0.0001. ns, not significant; PTC-209 (PTC); and Frondoside-A (Fr).

We demonstrate that treatment of the cells for 48 h with PTC-209 (1 μM) abbreviated PTC (1) enhances the anti-cancer effects of cisplatin (1 μM) in LNM35 lung cancer cells (**Figure 7A**) and cisplatin (5 μM) in A549 lung cancer cells (**Figure 7C**). However, PTC (1) failed to enhance the anti-cancer effect of cisplatin (1 μM) in A549 lung cancer cells (**Figure 7B**). PTC (1) was also able to enhance the anti-cancer effects of camptothecin (0.5 μM) in breast cancer cells, MDA-MB-231 (**Figure 7D**). However, PTC (1) failed to enhance the effects of oxaliplatin (1 μM) and 5-fluorouracil (0.5 μM) in colon cancer cell, HT-29 (**Figures 7E, F**). On the other hand, treatment of the cells for 48 h with PTC (1) significantly enhance the inhibitory effects, on cell viability, of different concentration of Frondoside-A (0.5, 1, and 2.5 μM) and in all the six cell lines tested (**Figures 8A–F**).

## Discussion

In the present study, we tried to decipher the mechanism of action PTC-209 and to investigate its potential anticancer activity alone and in combination with a major natural molecule, frondoside-A, and with several chemotherapeutic anti-cancer drugs. Our findings demonstrate that PTC-209 reduced the viability, colony, and tumor growth of a broad spectrum of cancer cell lines (lung, colon, and breast) through the inhibition of cellular proliferation and the induction of cell death. Interestingly, we provide evidence that PTC-209 mediates its anticancer effect, at least in part, through downregulation of the STAT3 signaling pathway. Furthermore, we show for the first time that PTC-209 at very low concentration of 0.01 and 0.1 µM inhibited the migratory potential of lung and breast cancer cells. In clinical practice, there is a growing interest in combination therapy using multiple drugs targeting different targets/pathways to improve the efficacy of the treatments and to decrease their potential side effects. In this context, we show that, when used in combination, PTC-209 significantly enhanced the anticancer activity of cisplatin, camptothecin, and frondoside A.

The anticancer activity of PTC-209, a specific inhibitor of Bmi-1 proto-oncogene and a member of the polycomb repressor complex 1, has been widely investigated in recent years. PTC-209 was shown to reduce cellular viability through inhibition of cellular proliferation and induction of apoptotic cell death in various cancers, including colorectal cancer (Kreso et al., 2014), head neck squamous cell carcinoma “HNSCC” (Wang et al., 2017), MM (Bolomsky et al., 2016; Alzrigat et al., 2017), BTC (Mayr et al., 2016), and acute myeloid leukemia (Nishida et al., 2015). In MM cells, PTC-209 was shown to reduce cell viability through induction of apoptosis measured by the activation of the executioner caspase 3/7 (Alzrigat et al., 2017). On the other hand, reduced cell viability of HNSCC, acute myeloid leukemia, BTC cells, and MM by PTC-209 was associated with G1 arrest and with an increase in caspase activity (Nishida et al., 2015; Bolomsky et al., 2016; Mayr et al., 2016; Wang et al., 2017). This has also been confirmed by Srinivasan group who reported that PTC-209 decrease mouse breast cancer stem cells proliferation by arresting the cells at the G0/G1 stage (Srinivasan et al., 2017). This suggests that PTC-209 effect on cell viability of BTC might be a result of inhibition of cellular proliferation and that apoptosis accounted only a little. Interestingly, in the ovarian cancer cells CP20 and OVCAR4, PTC-209 reduced cellular viability through induction of a non-apoptotic, caspase-independent autophagic cell death (Dey et al., 2016). Our data showed that PTC-209 inhibited cellular proliferation and induced cell death in caspase 3/7-independent manner.

Reports showed that PTC-209–induced cell death is associated with the downregulation of Bmi-1. Although it has been widely reported that PTC-209 exerts its activity through downregulation of Bmi-1 protein level in various types of cancer cells (Kreso et al., 2014; Bolomsky et al., 2016; Yong et al., 2016; Alzrigat et al., 2017; Wang et al., 2017), there are still controversy regarding the exact mechanism through which it downregulates Bmi-1. In head–neck squamous cell carcinoma Cal27 and FaDu cell lines, PTC-209 seems to downregulate Bmi-1 expression both at transcription and posttranslational levels. Indeed, PTC-209 not only significantly reduced the level of Bmi-1 transcript in the HNSCC but also targeted Bmi-1 protein to proteasome degradation (Wang et al., 2017). In MM cell lines, on the other hand, PTC-209 treatment downregulated Bmi-1 protein level but strikingly led to an increase in Bmi-1 transcript levels (Alzrigat et al., 2017). Interestingly, proteasome inhibition in MM (RPMI-8226 and LP-1) cell lines failed to rescue the Bmi-1 protein levels, suggesting that PTC-209 is an inhibitor of Bmi-1 translation (Alzrigat et al., 2017). PTC-209 at a concentration of 2.5-µM decrease Bmi-1 protein expression by about 30% as reported in FMMC 419II mouse mammary tumor cells (Srinivasan et al., 2017). It has been reported that PTC-209 at a similar concentration used in our study was able to decrease Bmi-1 protein expression in both MDA-MB-231 and MCF-7 breast cancer cells (Dimri et al., 2016) and A549 lung cancer cells (Yong et al., 2016). It has also been reported that the basal Bmi-1 levels may not be a determinant of PTC-209 sensitivity at least in acute myeloid leukemia (Nishida et al., 2015).

It is legitimate to hypothesize that PTC-209 might have a target(s) other than Bmi-1, which is yet to be identified, because of the impact of PTC-209 on Bmi-1 transcription. Constitutive activation of the transcription factor STAT3 has been reported in different types of cancers, including lung, breast, and colon. The crucial role of STAT3 in tumor cell survival, proliferation, migration, invasion, and metastasis is well established and, thus, it is targeting for therapy assaults cancer on multiple fronts (Rivat et al., 2005; Al Kubaisy et al., 2016; Aryappalli et al., 2017). Interestingly, our data clearly show that PTC-209 inhibits STAT3 signaling pathway through a decrease of STAT3 phosphorylation. This suggests that the anticancer effect of PTC-209 on cancers cells involves, although maybe not solely, the inactivation of the STAT3 signaling Pathway. This inhibition of STAT3 phosphorylation by PTC-209 can be due to the inhibition of upstream signaling targets, including the non-receptor tyrosine kinase c-SRC, the JAK2 tyrosine kinase, or the gp130 subunit of the IL-6 receptor (Groner, 2012; Soutto et al., 2019). Secretion of IL-6 by tumor cells and tumor microenvironment cells contributes to STAT3 activation. Indeed, binding of IL-6 to the membrane-bound IL-6R recruits a dimer of two gp130 and forms the active signaling complex, IL-6/IL-6R/gp130 complex, ultimately leading to the phosphorylation and activation of STAT3. Likewise, STAT3 activation induces IL-6 gene transcription and, thus, establishes a feedback loop in tumor tissues (Groner, 2012; Soutto et al., 2019).

Interestingly, our data show for the first time that PTC-209, a selective inhibitor of BMI-1, inhibits gp130-STAT3 pathway in cancer cells. Further investigations are needed to clarify the mechanism (s) by which PTC-209 inhibits STAT3 phosphorylation.

Recent studies reported that PTC-209 potentiates the anticancer activity of several clinically used drugs. Indeed, PTC-209 was shown to synergize the activity of cisplatin in the gallbladder (GBC) (Mayr et al., 2016) and HNSCC (Wang et al., 2017) cancer cells lines, respectively. Also, when combined with UNC1999, an inhibitor of EZH2 or JQ1, a BET bromodomain inhibitor, PTC-209 exhibited both synergistic and additive effects in MM (INA-6, JJN3, RPMI-8226, and LP-1) cancer cell lines (Alzrigat et al., 2017). In consistency with these reports, we show that PTC-209 enhance the anticancer activities of two major chemotherapeutic drugs namely cisplatin, and camptothecin. In addition, PTC-209 significantly enhance the anti-cancer effect of frondoside-A, a natural triterpenoid glycoside with anticancer activity against various type of cancer (Al Marzouqi et al., 2011; Attoub et al., 2013; Al Shemaili et al., 2014; Attoub et al., 2018), in lung (LNM35 and A549), breast (MDA-MB-231), and colon (HT-29 and HCT-116) cancer cells lines. This study provides a sufficient rationale to further carry out animal studies to confirm the relevance of the combination therapy using PTC-209. We believe that it may improve solid cancer therapy in combination with cisplatin and frondoside-A.

In summary, we demonstrate for the first time that PTC-209 inhibits STAT3 signaling pathway, leading to the inhibition of cellular proliferation and the induction of cellular death, consequently decreasing tumor growth. We also demonstrate the potential value of combining PTC-209 with major chemotherapeutic drugs, namely, cisplatin and camptothecin and with the natural compound frondoside-A, a marine molecule isolate from the sea cucumber *Cucumaria Frondosa*.

## Data Availability Statement

All datasets generated for this study are included in the manuscript/supplementary files.

## Ethics Statement

The in ovo LNM35 xenografts assay was done according to the protocol approved by the animal ethics committee at the United Arabs Emirates University. The in ovo A549 xenografts assay was done by INOVOTION company in France. According to the European Directive 2010/63/EU on protection of animals used for scientific purposes and French Regulations (Code Rural R214-89 to R214-137, last modification in 2013) which cover the use of chicken embryos at day 18 post-fertilization or later, there are no ethic constraints because our studies are stopped at day 18 of the embryo development (E18). Moreover, the Animal Experimentation Ethical Committee of Grenoble area has validated that we don’t need the IACUC approvals for our assays.

## Author Contributions

SA conceived and designed the experiments; KA and SS performed the experiments; SA and RI analysed and wrote the paper. All authors contributed to manuscript revision, read and approved the submitted version.

## Funding

This work was supported by the CMHS grant (31M275) and partly supported by the Terry Fox UAE grant (21M081). The funders had no role in study design, data collection and analysis, decision to publish, or preparation of the manuscript.

## Conflict of Interest

The authors declare that the research was conducted in the absence of any commercial or financial relationships that could be construed as a potential conflict of interest.
